# FKBP3 Induces Human Immunodeficiency Virus Type 1 Latency by Recruiting Histone Deacetylase 1/2 to the Viral Long Terminal Repeat

**DOI:** 10.1128/mBio.00795-21

**Published:** 2021-07-20

**Authors:** Xinyi Yang, Xiaying Zhao, Yuqi Zhu, Yinzhong Shen, Yanan Wang, Panpan Lu, Zhengtao Jiang, Hanyu Pan, Jinlong Yang, Jingna Xun, Lin Zhao, Jing Wang, Zhiming Liang, Xiaoting Shen, Yue Liang, Qinru Lin, Huitong Liang, Lu Jin, Dengji Zhang, Jun Liu, Bin Wang, Shibo Jiang, Jianqing Xu, Hao Wu, Hongzhou Lu, Huanzhang Zhu

**Affiliations:** a State Key Laboratory of Genetic Engineering and Engineering Research Center of Gene Technology, Ministry of Education, Institute of Genetics, School of Life Sciences, Fudan University, Shanghai, China; b Department of Infectious Disease, Key Laboratory of Medical Molecular Virology of Ministry of Education/Health, School of Basic Medical Sciences and Shanghai Public Health Clinical Center, Fudan University, Shanghai, China; c Center for Infectious Diseases, Beijing You'an Hospital, Capital Medical University, Fengtai District, Beijing, China; University of Pittsburgh; University of Pittsburgh School of Medicine

**Keywords:** HIV-1, HIV-1 latency, FKBP3, HDAC1/2, acetylation

## Abstract

Human immunodeficiency virus type 1 (HIV-1) cannot be completely eliminated because of existence of the latent HIV-1 reservoir. However, the facts of HIV-1 latency, including its establishment and maintenance, are incomplete. FKBP3, encoded by the *FKBP3* gene, belongs to the immunophilin family of proteins and is involved in immunoregulation and such cellular processes as protein folding. In a previous study, we found that FKBP3 may be related to HIV-1 latency using CRISPR screening. In this study, we knocked out the *FKBP3* gene in multiple latently infected cell lines to promote latent HIV-1 activation. We found that FKBP3 could indirectly bind to the HIV-1 long terminal repeat through interaction with YY1, thereby recruiting histone deacetylase 1/2 to it. This promotes histone deacetylation and induces HIV-1 latency. Finally, in a primary latent cell model, we confirmed the effect of *FKBP3* knockout on the latent activation of HIV-1. Our results suggest a new mechanism for the epigenetic regulation of HIV-1 latency and a new potential target for activating latent HIV-1.

## INTRODUCTION

Antiretroviral therapy for AIDS patients can decrease viral plasma load in the blood below the detection limit (1, 2). However, once the patient stops antiretroviral therapy, the plasma load rebounds rapidly (3, 4). This is primarily caused by the virus reservoir composed of latent human immunodeficiency virus type 1 (HIV-1) infection and long-term resting cells (5–7). In the last few decades, studies have clarified the molecular mechanisms underlying the establishment of HIV-1 latency, mostly acting at the level of transcriptional suppression of the viral promoter-long terminal repeats (LTR) (8–13). Additionally, researchers have found that HIV-1 transcriptional blocks are related to multiple layers of regulation. This includes epigenetic modifications at the HIV-1 LTR, including EZH2 (14, 15), histone deacetylase 1 and 2 (HDAC1/2) (16–18), SUV39H1 (19), chromatin repressors, and inadequate availability of transcription factors (TFs) at the HIV LTR, such as NF-κB (20–22), positive transcription elongation factor b (P-TEFb or CDK9/CycinT1) (23, 24), and HIV-1 Tat (20, 25), among others (26–28). Researchers have also identified a variety of latency reversal agents (LRAs) that are functional *in vitro.* However, in spite of these advances, no LRA can significantly reduce the virus reservoir *in vivo*, indicating the limitations of our current understanding of the mechanisms underlying HIV-1 establishment and maintenance (29, 30).

Recently, with the emergence of gene-editing technology, especially the CRISPR/Cas9 system, methods for investigating gene function have been greatly expanded (31–34). In our previous research, we screened host genes related to HIV-1 latency using a CRISPR library, and identified many potential candidate genes related to HIV-1 latency, including *UBB*, *SERBP1*, *ZDHHC1*, *CNTNAP1*, *PEBP1*, and *FKBP3* (35). Among the enriched genes, we determined that PEBP1 induces HIV-1 latency by inactivating the MAPK and IKK signaling pathways and inhibiting NF-κB entry into the nucleus. In the current study, we further identified *FKBP3* associated with the suppression of HIV replication and promotion of HIV latency.

Only a few studies on FKBP3 have been reported, and its function is not fully understood. FKBP3 (also known as FKBP25) is a member of the FK506 binding protein (FKBP) family (36). It contains two functional domains, the peptidylprolyl *cis*-*trans* isomerase domain and the domain binding to immunosuppression (37). FKBP3 is mainly located in the nucleus and is a molecular chaperone of the p53 regulatory protein MDM2 that can regulate the expression of p53 and p21 (38). However, the relationship between FKBP3 and HIV-1 latency has not yet been reported.

In the present study, when *FKBP3* was knocked out, latent HIV-1 was reactivated in multiple latent cell lines and primary CD4-positive (CD4^+^) latent T cell models. Importantly, we confirmed that FKBP3 could indirectly bind to the HIV-1 LTR through interaction with YY1, thereby recruiting HDAC1/2 to the HIV-1 LTR. This promotes histone deacetylation and induces HIV-1 latency. We also found that when HIV-1 infects T lymphocytes, the expression level of FKBP3 was affected by interferon beta (IFN-β) and IFN-γ. To the best of our knowledge, this is the first instance of linking the *FKBP3* gene and HIV-1 latency via epigenetic gene modification. Thus, our study presents mechanistically novel insights into the molecular mechanism of HIV latency that could facilitate development of a novel target for therapeutic intervention against HIV latency.

## RESULTS

### Knocking out the *FKBP3* gene can activate latent HIV-1 in multiple latently infected cell models.

**FIG 1:**
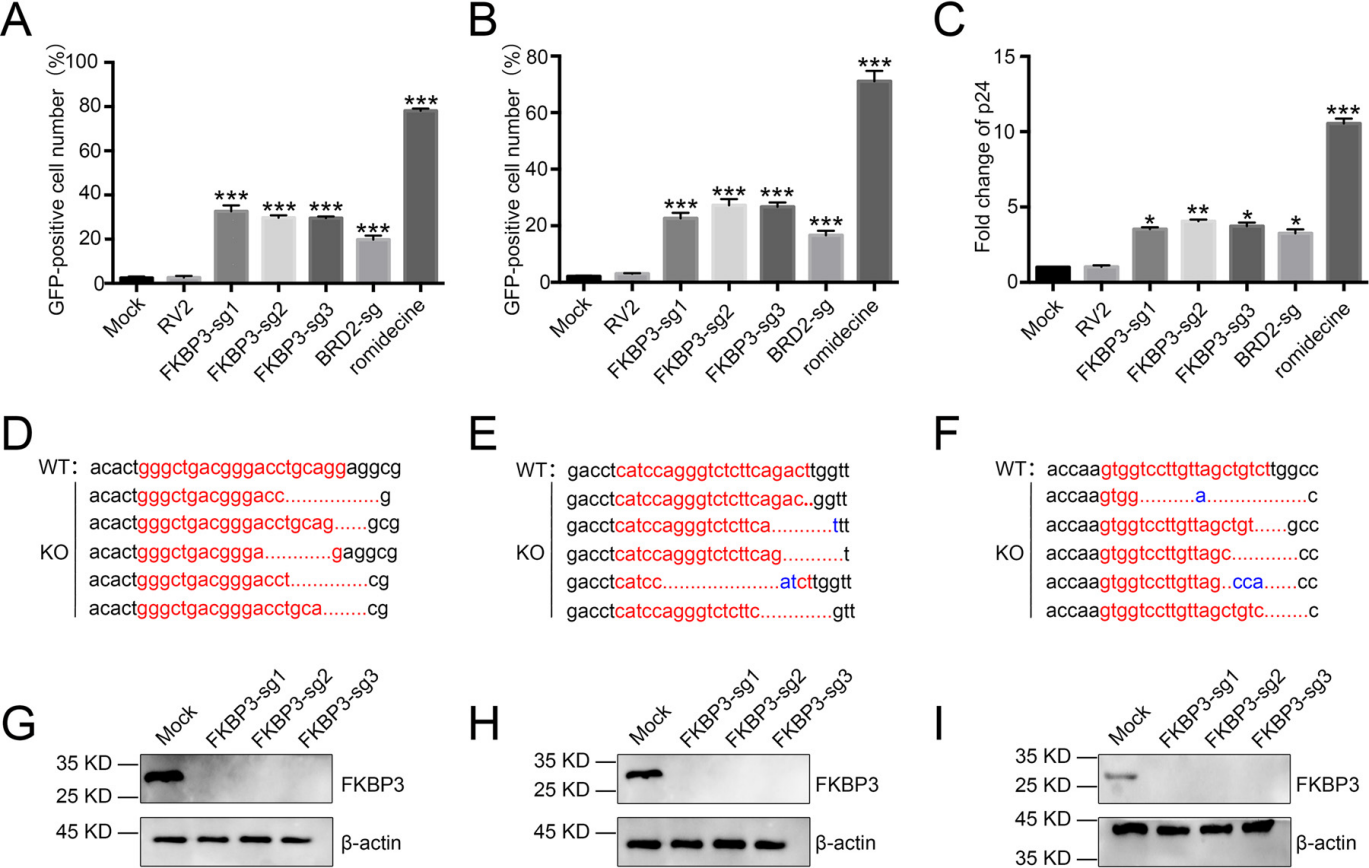
Knocking out the *FKBP3* gene can promote HIV-1 latent reversal in HIV-1 latently infected cell lines. (A and B) Knocking out the *FKBP3* gene can promote HIV-1 latent reversal in C11 and J-Lat 10.6 cell lines. C11 and J-Lat 10.6 cell lines were infected by lentiCRISPR v2.0 packaged lentiviruses with sgRNA, followed by screening for 14 days with purinomycin 2 μg/ml. The percentage of GFP-positive cells was measured by flow cytometry to determine the level of HIV-1 reactivation. Each datum represented the mean ± SD of three independent experiments (*n *= 3) and was analyzed with *t* test. *, *P < *0.05; **, *P < *0.01; ***, *P < *0.001. (C) The effect of knocking out the *FKBP3* gene on HIV latency was further verified in ACH2 models of HIV latency. The expression levels of p24 in ACH2 cells were detected by HIV-1 p24 ELISA. Each datum represented the mean ± SD of three independent experiments. (*n *= 3) and was analyzed with *t* test. *, *P < *0.05; **, *P < *0.01; ***, *P < *0.001. (D to F) Sequencing FKBP3 PCR products after clone screening. The PCR products of *FKBP3* gene were cloned and then sequenced. FKBP3-sg1 (D), FKBP3-sg2 (E), and FKBP3-sg3 (F) target sites are shown in red letters. Dashes indicate the deleted bases relative to the wild-type sequence. (G to I) FKBP3 protein levels were measured by Western blotting after knockout in C11 (G), J-Lat 10.6 (H), and ACH2 (I) cells by LvFKBP3-sg1, LvFKBP3-sg2, LvFKBP3-sg3, and mock C11 cells, which served as control.

In our previous research, we identified many genes that may be related to HIV-1 latency using CRISPR screening, one of which was *FKBP3* (35). Subsequently, we explored whether the enriched candidate gene *FKBP3* was actually involved in HIV-1 latency through CRISPR library screening. To validate the relationship between *FKBP3* and HIV-1 latency, we infected C11 cells as an HIV latency model. These cells were previously established in our laboratory. They harbor an HIV-1 proviral DNA with a reporter gene that encodes green fluorescent protein (GFP) (39, 40), with CRISPR/Cas9 with specific guide RNAs (sgRNAs) targeting *FKBP3* or without sgRNA lentivirus followed by selection with puromycin (2 μg/ml) treatment for 14 days. *FKBP3* knockout significantly induced reactivation of latent HIV-1 by approximately 30% (Fig. 1A). To further confirm the correlation between FKBP3 and HIV-1 latency, we tested two other latent cell models, including J-Lat 10.6 and ACH2 cell lines, and observed similar effects (Fig. 1B and C). To prove that *FKBP3* was indeed knocked out in the latency-disrupted C11 cells, we sequenced the genomic targeting sites in the potential *FKBP3* knockout cell clones using genomic DNA sequencing. *FKBP3* was deleted in the target sites of *FKBP3* sgRNA1 to sgRNA3 with different forms of indels (Fig. 1D to F). FKBP3 was nearly undetectable after knockout of *FKBP3* targeted with *FKBP3* sgRNAs in all latency model cell lines (Fig. 1G to I). Taken together, our data suggest that *FKBP3* is a new HIV latency-associated gene.

### FKBP3 indirectly binds to HIV-1 LTR through interaction with YY1.

**FIG 2:**
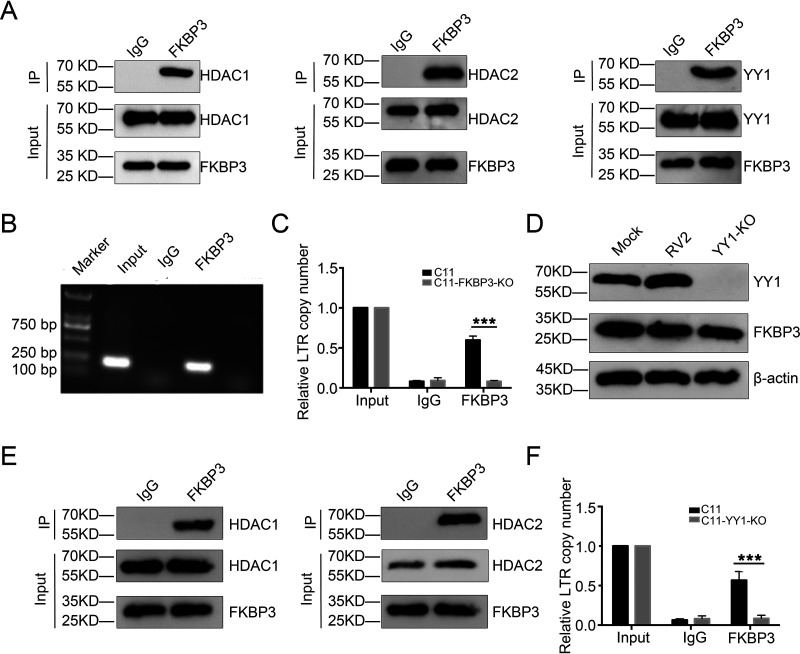
FKBP3 could interact with YY1 and HDAC1/2 and indirectly bind to the HIV-1 LTR site through YY1 in an HIV-1 latently infected cell line. (A) The immunoprecipitation assay was performed in C11 cell lysates with anti-FKBP3 antibody, followed by Western blotting with anti-YY1, anti-HDAC1, or anti-HDAC2 antibodies. (B and C) The interaction between FKBP3 and HIV-1 LTR was detected by ChIP. Chromatin fragments from C11 cells and C11-FKBP3-KO cells were immunoprecipitated with anti-FKBP3 antibodies or control normal rabbit serum (IgG). After ChIP, the binding of FKBP3 to HIV LTR was detected by PCR (B) and qPCR (C) with specific primers for HIV LTR. The number of copies was normalized to input group. Each datum represented the mean ± SD of three independent experiments (*n *= 3) and was analyzed with *t* test. ***, *P < *0.001. (D) Identification of the lack of YY1 and FKBP3 protein expression in C11-YY1-KO cells. YY1 and FKBP3 protein level was measured by Western blotting after knockout in C11 cells by lentivirus sgRNA-YY1. Mock C11 cells and lentiCRISPR v2.0 without sgRNA (RV2)-infected C11 cells served as mock control. (E) The interaction between FKBP3 and HIV-1 LTR was detected by ChIP in the C11-YY1-KO cell line. Chromatin fragments from C11 cells and C11-YY1-KO cells were immunoprecipitated with anti-FKBP3 antibodies or control normal rabbit serum (IgG). After ChIP, the binding of FKBP3 to HIV LTR was detected by qPCR with specific primers for HIV LTR. The number of copies was normalized to input group. Each datum represented the mean ± SD of three independent experiments and was analyzed with *t* test. ***, *P < *0.001.

FKBP3 is a rapamycin binding receptor (36–38) and can also act as an adaptor to interact with the YY1 protein, HDAC1/2 (41). Furthermore, HDAC1/2 and YY1 are related to HIV-1 latency, and HDAC inhibitors can disrupt latent HIV-1 (42–44). Therefore, we speculated that FKBP3 is involved in HIV-1 latency through its interaction with YY1 and HDAC1/2. To test this speculation in HIV-1 latent cells, we performed a coimmunoprecipitation (co-IP) assay and found that FKBP3 interacted with both YY1 and HDAC1/2 in the C11 HIV latency cell model (Fig. 2A). Bioinformatics research previously found that FKBP3 could also interact with DNA in cells. Therefore, to verify interaction between FKBP3 and the HIV-1 genome in cells, we performed chromatin immunoprecipitation (ChIP) and quantitative PCR (qPCR). We found that FKBP3 could, indeed, bind to the HIV-1 LTR (Fig. 2B and C). However, since the YY1 protein binds to the HIV-1 LTR (18), we reasoned that FKBP3 might also bind to HIV-1 LTR through indirect binding to YY1 or by direct binding to FKBP3. To this end, we constructed YY1 knockout latently infected cells (Fig. 2D). *YY1* knockout caused no changes in FKBP3 levels (Fig. 2D). Therefore, we subsequently repeated the ChIP assay, and when YY1 was deleted, we could not significantly enrich the HIV-1 LTR with anti-FKBP3, indicating that FKBP3 does, indirectly, bind to the HIV-1 LTR through YY1 (Fig. 2E and F).

### FKBP3 recruits HDAC1/2 to HIV-1 LTR to promote histone deacetylation.

**FIG 3:**
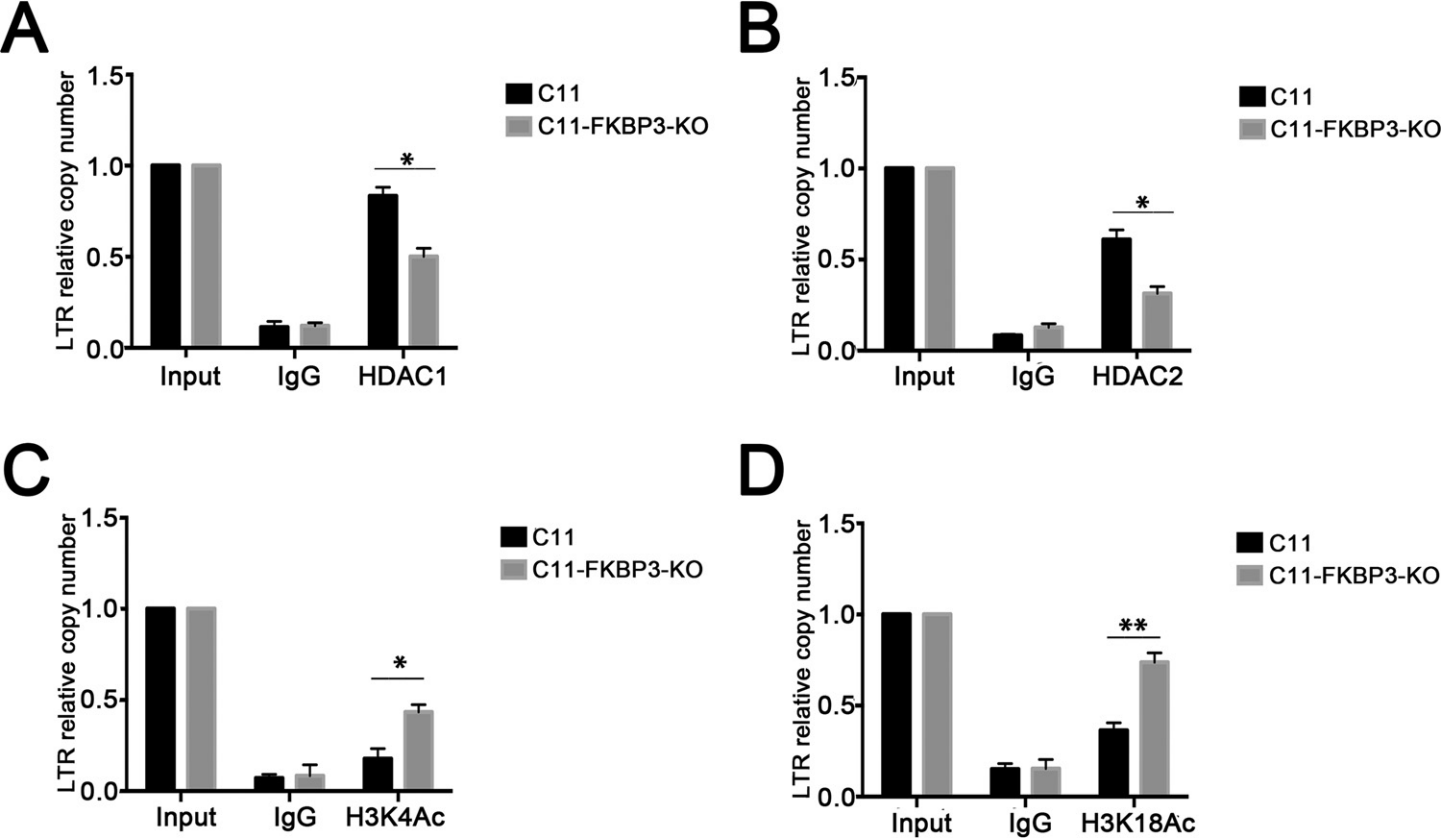
FKBP3 promotes histone deacetylation by recruiting HDAC1/2 to HIV-1 LTR. (A and B) The effect of *FKBP3* knockout on HDAC1 (A) or HDAC2 (B) protein recruitment into HIV LTR was detected by ChIP. Chromatin fragments from C11 cells and C11-FKBP3-KO cells were immunoprecipitated with HDAC1 (A) or HDAC2 (B) antibodies or control normal rabbit serum (IgG). After ChIP, the binding of HDAC1 (A) or HDAC2 (B) to HIV LTR was detected by qPCR with specific primers for HIV LTR. The number of copies was normalized to input group. Each datum represented the mean ± SD of three independent experiments and was analyzed with *t* test. *, *P < *0.05. (C and D) The effect of *FKBP3* knockout on histone acetylation of HIV LTR was detected by ChIP. Chromatin fragments from C11 cells and C11-FKBP3-KO cells were immunoprecipitated with H3K4Ac (C) or H3K18Ac (D) antibodies or control normal rabbit serum (IgG). After ChIP, the histone acetylation of H3K4Ac (C) or H3K18Ac (D) of HIV LTR was detected by qPCR with specific primers for HIV LTR. The number of copies was normalized to input group. Each datum represent the mean ± SD of three independent experiments (*n *= 3) and was analyzed with *t* test. *, *P < *0.05; **, *P < *0.01.

Although FKBP3 can indirectly bind to HIV-1 LTR via YY1, its biological function in HIV-1 latency remained unclear. Given that FKBP3 could also interact with HDAC1/2, we considered that FKBP3 might recruit HDAC1/2 to the HIV-1 LTR, thereby promoting histone deacetylation. To test our hypothesis, we first knocked out *FKBP3* and confirmed that the expression of HDAC1/2 in latently infected cells was not significant (Fig. S1A in the supplemental material). We then detected the relative amount of HDAC1/2 bound to the HIV-1 LTR in C11 and C11-FKBP3-knockout (KO) cells using ChIP and qPCR. In either HDAC1 or HDAC2, when *FKBP3* is knocked out, we found binding to the HIV-1 LTR to be relatively reduced (Fig. 3A and B). The biological function of HDAC1/2 has been very clear. Therefore, we decided to further explore changes in acetylation of histone sites H3K4 and H3K18 related to HIV-1 latency. When *FKBP3* was knocked out in latent cells, acetylation of H3K4 and H3K18 in histones near the HIV-1 LTR increased (Fig. 3C and D). However, the total protein acetylation of H3K4 and H3K18 in these cells did not change significantly (Fig. S1B). Importantly, these data indicate that FKBP3 promotes histone deacetylation by recruiting HDAC1/2 to the HIV-1 LTR, thereby inducing HIV-1 latency.

10.1128/mBio.00795-21.1 FIG S1 Effect of FKBP3 knockout on the expression of related genes. (A) Effect of FKBP3 knockout on HDAC1 and HDAC2. The levels of indicated proteins in total protein lysates were measured with Western blot analysis in C11-FKBP3-KO cells and mock C11 cells. (B) Effect of FKBP3 knockout on the acetylation of H3K4 and H3K18. The levels of indicated proteins in nucleus were measured with Western blot analysis in C11-FKBP3-KO cells and mock C11 cells. Download FIG S1, TIF file, 0.6 MB. Copyright © 2021 Yang et al. 2021 Yang et al. https://creativecommons.org/licenses/by/4.0/ This content is distributed under the terms of the Creative Commons Attribution 4.0 International license.

### FKBP3 induces HIV latency in primary CD4^+^ T cells.

**FIG 4:**
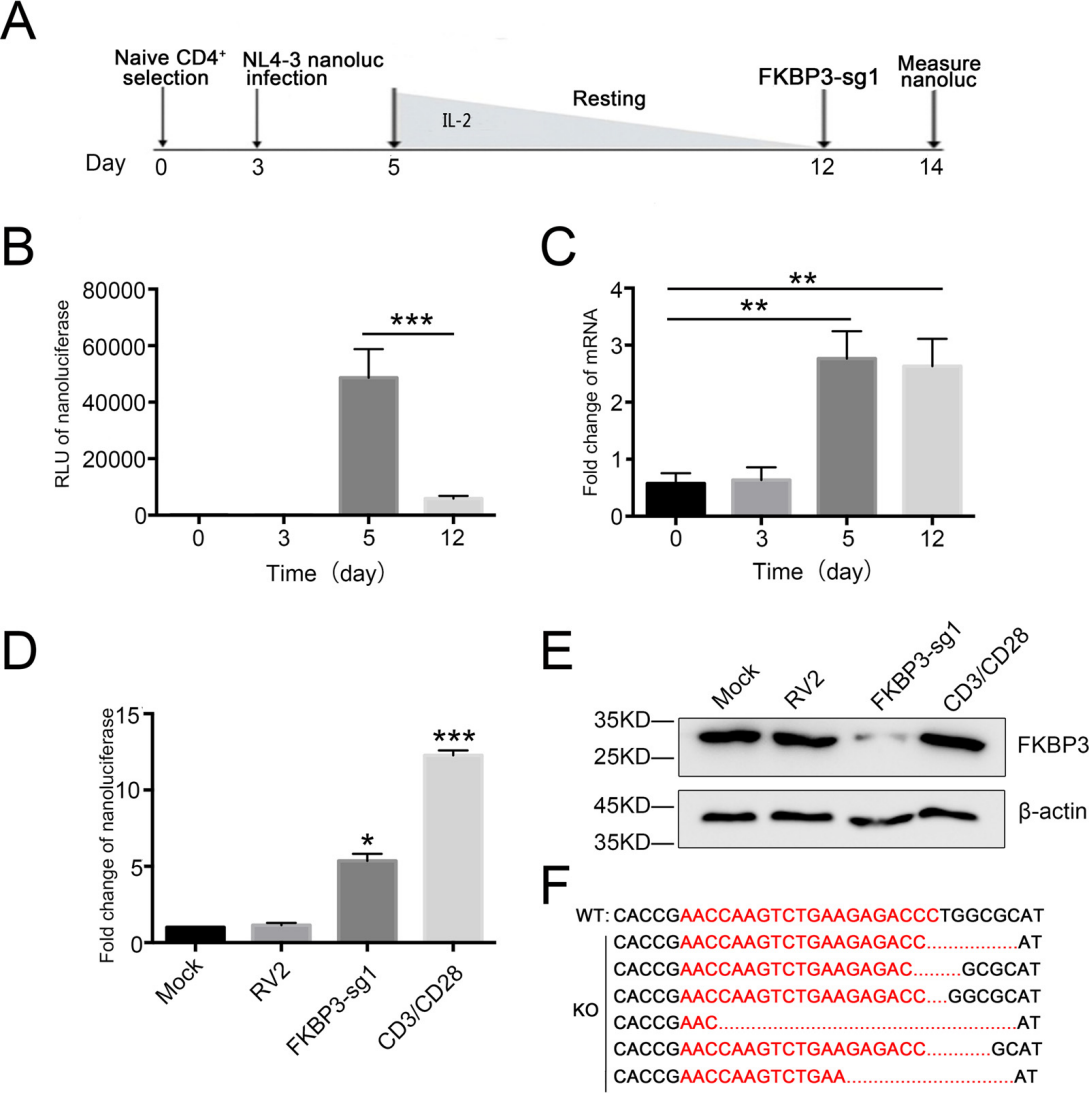
Knockout FKBP3 reactivates latent HIV-1 in the primary CD4^+^ T model of latency (3 donors). (A) Outline of the protocol to establish HIV latency. Human primary CD4^+^ T cells were activated and expanded with α-CD3/CD28 beads on day 1. The α-CD3/CD28 beads were removed on day 3. Cells were then infected with HIV-1 NL4.3-NanoLuc on the 3rd day after expansion and maintained over 7 days with a decreasing concentration of IL-2 to establish latency until day 12. On the 12th day, cells were transduced with Cas9 and FKBP3-sgRNA plasmid by electroporation. (B) During HIV-1 infection, the transcription of HIV-1 in the primary CD4^+^ T cells was determined by NanoLuc luciferase assay at different time points. Each datum represented the mean ± SD of three independent experiments and was analyzed with *t* test. ***, *P < *0.001, compared with the cells 5 days postinfection. (C) After infection with vesicular stomatitis virus glycoprotein G (VSV-G)-pseudotyped HIV-1 NL4.3-NanoLuc, the mRNA expression of FKBP3 was measured by qPCR. Each datum represented the mean ± SD of three independent experiments and was analyzed with *t* test. **, *P < *0.01, compared with uninfected mock cells. (D) The role of FKBP3 in the primary CD4^+^ T cell model of latency. The expression of HIV-1 was measured by NanoLuc in the primary CD4^+^ T cells after gene knockout by mock, RV2, and FKBP3-sg1 with or without α-CD3/CD28 stimulation. Each datum represented the mean ± SD of three independent experiments and was analyzed with *t* test. *, *P < *0.05; ***, *P < *0.001, compared with cells after Lv-RV2 mock knockout. (E) FKBP3 protein was determined by Western blot of whole-cell lysate from primary CD4^+^ T cells. (F) Sequencing FKBP3 PCR products after Lv-FKBP3-sg1 knockout in primary CD4^+^ T cells. The PCR products of FKBP3 were cloned and then sequenced. FKBP3-sg1 target sites are shown in red letters. Dashes indicate deleted bases relative to the wild-type sequence.

Our data support the hypothesis that FKBP3 is a latency-inducing protein. To directly test whether FKBP3 promotes HIV-1 latency in primary human CD4^+^ T lymphocytes, we established a primary HIV-1 latent infection model as described before (45, 46) (Fig. 4A) (primary CD4^+^ T lymphocyte cells from three donors). NanoLuc luciferase expression was detected 2 days post-HIV-1 infection, indicating active HIV infection. Twelve days after a gradual decrease in interleukin-2 (IL-2) level, a significant decrease in NanoLuc luciferase expression was observed (Fig. 4B), indicating that HIV-1 was in a state of latency in these primary CD4^+^ T cells. Importantly, when HIV-1 infected the primary CD4^+^ T lymphocytes, the expression level of FKBP3 in these cells increased significantly, suggesting a possible involvement in the immune response of CD4^+^ T lymphocytes to HIV-1 infection (Fig. 4C).

Subsequently, in the established primary CD4^+^ T lymphocyte latent model, we knocked out *FKBP3* by targeting FKBP3 of Cas9-sgRNA using electroporation, and we detected the expression level of NanoLuc luciferase. In the primary latent model, *FKBP3* knockout had an activation effect similar to that in latently infected cell lines (Fig. 4D). To prove that *FKBP3* was indeed knocked out in primary CD4^+^ T lymphocyte cells, we sequenced the genomic targeting sites in these *FKBP3* knockout cells using genomic DNA sequencing and detected the changes at the FKBP3 level. We found that *FKBP3* was deleted at the target sites of *FKBP3* sgRNA1 with different forms of indels (Fig. 4E and F). Electroporation has been known to cause activation and death of primary cells, thereby affecting the activation of latent HIV-1 in cells. Therefore, to rule out false positives that may have been caused by experimental techniques, we tested the levels of cell activation and apoptosis in the knockout and control groups. Compared with the control group and the RV2 group, the expression level of the late T cell activation marker CD25 slightly increased in the cells of the FKBP3-KO group, but the expression level of the early activation marker CD69 did not change significantly. In addition, compared with the RV2 group, the apoptosis levels of cells did not change significantly in the FKBP3-KO group. In summary, we think that the activation of latent HIV-1 was primarily caused by *FKBP3* knockout (Fig. S2).

10.1128/mBio.00795-21.2 FIG S2 Effect of FKBP3 knockout on primary CD4+ T cells. (A) The expression of CD25 and CD69 was detected by flow cytometry using antibodies against CD25 and CD69 for mock primary CD4^+^ T cells and FKBP3-KO primary CD4^+^ T cells. (B) Apoptosis of FKBP3-KO primary CD4^+^ T cells and mock primary CD4^+^ T cells was measured by TUNEL staining followed by flow cytometry. Each datum represented the mean ± SD of three independent experiments and was analyzed with *t* test. Download FIG S2, TIF file, 1.6 MB. Copyright © 2021 Yang et al. 2021 Yang et al. https://creativecommons.org/licenses/by/4.0/ This content is distributed under the terms of the Creative Commons Attribution 4.0 International license.

### FKBP3 inhibits HIV-1 replication during viral infection.

**FIG 5:**
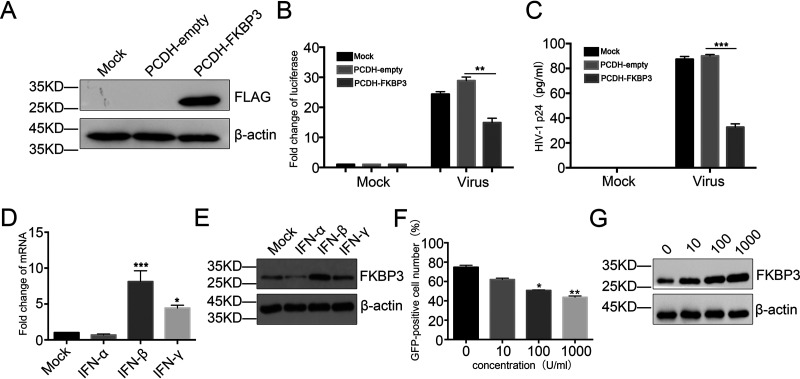
Effects of interferons on FKBP3 induction and FKBP3 inhibition of virus infection and replication. (A) The expression of FKBP3 protein was measured by Western blotting in TZM-bl cells infected with mock, Lv-PCDH-empty, or Lv-PCDH-PEBP1 virus. (B and C) The effect of overexpression of FKBP3 on HIV-1 replication. The cells were infected with supernatants of patient blood for 12 h and then washed three times. The transcription of HIV-1 was evaluated 72 h postinfection by luciferase activity (B) and levels of p24 released from supernatants (C). Each datum represented the mean ± SD of three independent experiments (*n *= 3) and was analyzed with *t* test. **, *P < *0.01; ***, *P < *0.001. (D and E) After treatment of IFN-α, IFN-β, and IFN-γ in Jurkat CD4^+^ T cells for 24 h, the mRNA (D) or protein (E) expression levels of FKBP3 were detected by qPCR or Western blot analysis. Each datum represented the mean ± SD of three independent experiments (*n *= 3) and was analyzed with *t* test. *, *P < *0.05; ***, *P < *0.001. (F) Ya cell lines were treated with different concentration of IFN-β for 48 h. The percentage of GFP-positive cells was measured by flow cytometry to determine the level of HIV-1 reactivation. Each datum represented the mean ± SD of three independent experiments (*n *= 3) and was analyzed with t-test. *, *P < *0.05; **, *P < *0.01. (E) FKBP3 protein was determined by Western blot of whole-cell lysate from Ya cells treated with IFN-β for 48 h.

The increased expression level of FKBP3 during establishment of the primary latent model inspired us to explore the role of FKBP3 in HIV-1 during the acute infection period. We first overexpressed FKBP3 in the TZM-bl cell line, which contains an LTR-driven luciferase reporter gene by lentivirus (Fig. 5A). We then infected TZM-bl cells that overexpressed FKBP3, or control, with virus in serum supernatants isolated from patients infected with HIV-1 for 12 h and washed the cells three times with phosphate-buffered saline (PBS). At 72 h postinfection, cells and supernatant media were harvested to measure HIV-1 transcription based on luciferase activity and p24 expression, respectively. The overexpression of FKBP3 could significantly inhibit the transcription and replication of HIV-1 (Fig. 5B and C). Therefore, during the acute infection period, cells expressed FKBP3 at a high level as an anti-HIV-1 response mechanism.

We next investigated how FKBP3 is induced during HIV-1 infection. Many factors involved in the restriction of HIV-1 transcription are related to IFN (47, 48). Therefore, we considered whether the change in FKBP3 expression during the acute infection by HIV-1 is also regulated by IFN. After treating Jurkat cells with different IFNs, we detected changes in FKBP3 expression using qPCR and Western blotting. When Jurkat cells were treated with IFN-β and IFN-γ, the expression of FKBP3 increased, and the effect of IFN-β on FKBP3 was more significant than that of IFN-γ (Fig. 5D and E). Given the effect of IFN-β on FKBP3, we asked if IFN-β could inhibit HIV-1 transcription and replication in an FKBP3-dependent manner. To address this question, we treated the Ya cell line where HIV-1 is active with different concentrations of IFN-β and found that the expression level of FKBP3 gradually increased as the concentration of IFN-β increased, whereas that of GFP, a marker of HIV-1 activation in Ya cells, gradually decreased (Fig. 5F and G). This indicates that IFN-β inhibits HIV-1 transcription and replication in an FKBP3-dependent manner.

### FKBP3 does not inhibit HIV-1 transcriptional activation through immune signaling pathways.

**FIG 6:**
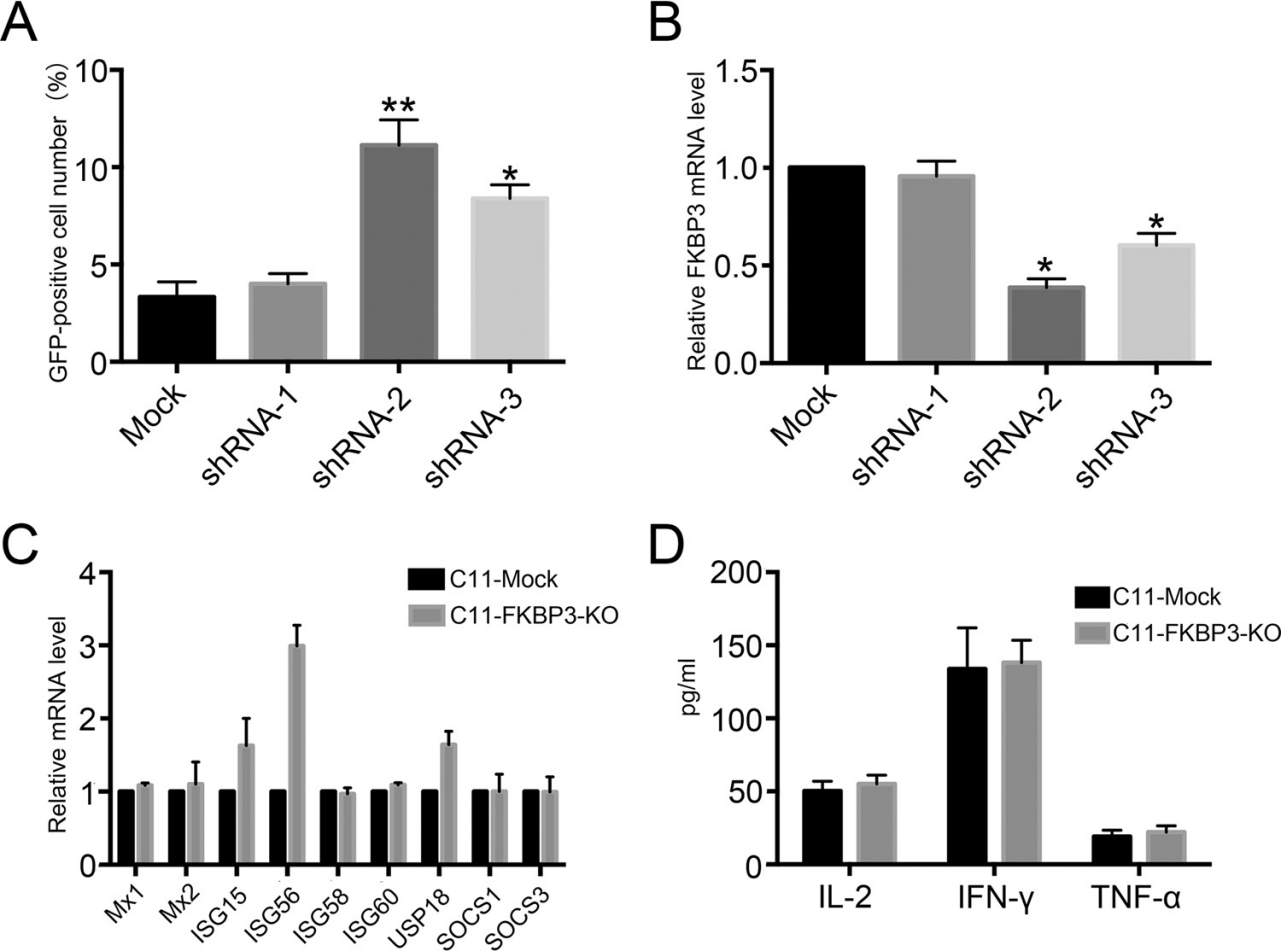
FKBP3 knockout does not cause significant changes in immune response. (A) Knocking down *FKBP3* could promote HIV-1 latent reversal in the C11 cell line. C11 cells were infected by pLKO.1 packaged lentivirus with shRNA targeting FKBP3. The percentage of GFP-positive cells was measured by flow cytometry to determine the level of HIV-1 reactivation. Each datum represented the mean ± SD of three independent experiments (*n *= 3) and was analyzed with *t* test. *, *P < *0.05; **, *P < *0.01; ***, *P < *0.001. (B) FKBP3 expression was measured by qPCR in C11 cells infected with lentivirus with or without shRNA targeting FKBP3. Each datum represented the mean ± SD of three independent experiments (*n *= 3) and was analyzed with *t* test. *, *P < *0.05; **, *P < *0.01; ***, *P < *0.001. (C) Expression of ISGs was measured by qPCR in C11 and C11-FKBP3-KO cells. Each datum represented the mean ± SD of three independent experiments (*n *= 3) and was analyzed with *t* test. *, *P < *0.05; **, *P < *0.01; ***, *P < *0.001. (D) The levels of IL-2, tumor necrosis factor alpha (TNF-α), and IFN-γ were evaluated by ELISA in the supernatants of C11 and C11-FKBP3-KO cells.

Considering that *FKBP3* is a potential interferon-stimulated gene (ISG), it may affect cell immune response. To determine whether the effect of FKBP3 on HIV-1 activation is mediated through the immune signaling pathway, we first tested the effect of *FKBP3* short hairpin RNA (shRNA)-mediated knockdown on the latent activation of HIV-1. Transient knockdown of FKBP3 could activate latent HIV-1 (Fig. 6A), and the efficiency of activation was related to the efficiency of shRNA in inhibiting FKBP3 (Fig. 6B). We further examined changes in the expression of nine selected ISGs and secretion of immune-related cytokines. Among the ISGs that we selected, only 3 ISGs showed changes in their expression levels, and no significant difference in the levels of two of them was observed (Fig. 6C). For cytokine secretion into the supernatant, no significant change occurred after *FKBP3* knockout (Fig. 6D). This indicates that FKBP3 does not promote HIV-1 latency by suppressing cellular immune responses.

### Knocking out *FKBP3* did not affect the proliferation and apoptosis of latent cells.

After confirming that FKBP3 is related to HIV-1 latency, we considered whether FKBP3 might become a latent activation target. The first condition for a gene to be a drug target is safety. Therefore, we preliminarily explored the situation of cell proliferation and apoptosis after *FKBP3* knockout using cell counting kit (CCK-8) and terminal deoxynucleotidyltransferase-mediated dUTP-biotin nick end labeling (TUNEL) staining. The proliferation rate was slightly higher in *FKBP3* knockout C11 cells than in control C11 cells, whereas *FKBP3* knockout did not affect apoptosis (Fig. S3A and B). Therefore, we believe that FKBP3 can potentially be used as a target for activation of latent HIV-1.

10.1128/mBio.00795-21.3 FIG S3 Effect of FKBP3 knockout on the proliferation and apoptosis of latently infected C11 cells. (A) Cell proliferation of FKBP3-KO-C11 cells and mock C11 cells was analyzed by CCK-8 levels. (B) Apoptosis of FKBP3-KO-C11 cells and mock C11 cells was measured by TUNEL staining followed by flow cytometry. Each datum represented the mean ± SD of three independent experiments and was analyzed with *t* test. Download FIG S3, TIF file, 0.2 MB. Copyright © 2021 Yang et al. 2021 Yang et al. https://creativecommons.org/licenses/by/4.0/ This content is distributed under the terms of the Creative Commons Attribution 4.0 International license.

## DISCUSSION

In this study, based on the data from our previous CRISPR library screening, we identified a previously unrecognized gene associated with HIV-1 latency, *FKBP3*. Our data revealed that FKBP3 could interact with YY1 and HDAC1/2 in latent cells and indirectly bind to the HIV-1 LTR through YY1, recruiting HDAC1/2 to promote deacetylation of histones near the HIV-1 LTR. This, in turn, induced HIV-1 latency. In addition, FKBP3 could be induced by IFN-β and IFN-γ during the acute infection period, thereby inhibiting HIV-1 transcription and replication.

**FIG 7:**
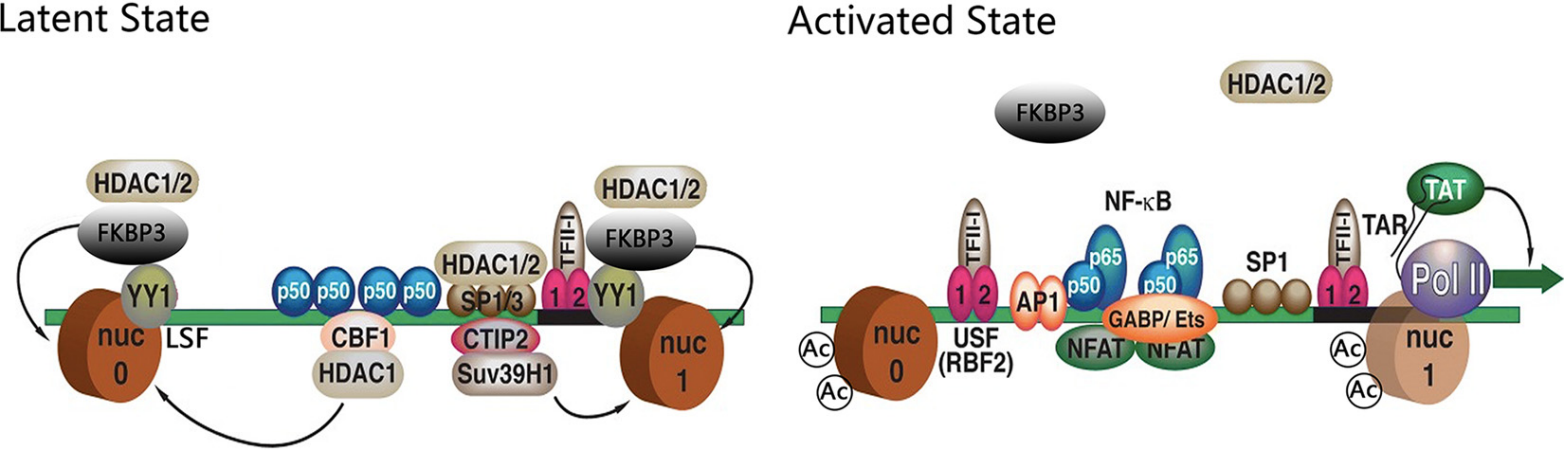
Working model of the role of FKBP3 in the establishment of HIV latency.

Based on our cumulative data, we propose a working model in which FKBP3 restricts HIV-1 transcription and is associated with HIV-1 latency (Fig. 7). When HIV-1 is in a latent state, FKBP3 can indirectly bind to the HIV-1 LTR via YY1 and recruit HDAC1/2 to promote histone deacetylation. When FKBP3 is eliminated, HDAC1/2 are not recruited to the HIV-1 LTR, and histone acetylation increases, which, in turn, promotes HIV-1 transcription.

Interestingly, HDAC1/2 can be recruited to the HIV-1 LTR by different proteins, including CTIP-2, NF-κB, and so on. Inhibitors of HDAC1/2, such as SAHA, M344, and chidamide, have been shown to activate latent HIV-1, and some of these inhibitors have progressed to clinical trials. In addition to histone acetylation, epigenetic modifications, such as histone methylation and crotonylation, have been shown to participate in HIV-1 latency. The reversibility of apparent modification has a high degree of overlap with the concept of HIV-1 latent activation. This suggests that epigenetic modification is an important factor in the establishment and maintenance of HIV-1 latency, thus addressing the gaps in our knowledge, as previously noted in this work.

In this study, we also found that *FKBP3* is a potential ISG. In the acute stage of HIV-1 infection, the secretion of cytokines, including IFN-β, is significantly increased. This promotes the immune response of cells, thereby inhibiting infection, replication, and transcription of the virus. An increase in the level of the immune response leads to significant changes in the expression of downstream ISGs, thereby changing the biological processes inside the cell. This protects the cell itself and increases its resistance to virus. Among the ISGs, some may also silence infecting viruses through epigenetic methods, similar to the action of *FKBP3* identified in this study. Therefore, the connection between immune response and epigenetics during the establishment and maintenance of HIV-1 latency is an interesting scientific research topic, but one for future studies.

We have only begun to understand the molecular mechanism by which FKBP3 promotes HIV-1 latency. Several questions remain to be solved. First, FKBP3 is a member of the FKBP family (36). The proteins of the FKBP family are potentially competitive binding receptors for rapamycin (49–51). Therefore, in HIV-1 latency, FKBP3 may also play a role by acting on the mammalian target of rapamycin (mTOR) signaling pathway in addition to affecting the HIV-1 latency through epigenetic inheritance. Second, FKBP3 is a molecular chaperone protein. It can stabilize p53 and Sp1, and Sp1 can also bind to the HIV-1 LTR (38). Therefore, the effect of interaction between FKBP3 and Sp1 on the HIV- 1 latency is also a factor to be considered in the future. Finally, there have been only a few reports on the biological functions of FKBP3. It is therefore unclear whether FKBP3 has unknown biological effects. Although we discovered the effect of FKBP3 on the latent phenotype of HIV-1, the molecular mechanisms underlying this effect are currently only partially understood, and further research is needed to understand this in detail.

In conclusion, we identified that FKBP3 suppresses HIV-1 transcription, which is essential for the establishment of HIV latency in CD4^+^ T cells by changing the state of histone acetylation and deacetylation. Considering that HDAC is an important latent activator of HIV-1 currently being investigated in the development of HIV cure strategies, this novel molecular mechanism of HDAC1/2 is particularly important.

## MATERIALS AND METHODS

### HIV-1 latently infected cell lines.

The HIV-1 latent infection model C11 cell line (constructed in our lab) (28, 35, 39) and J-Lat 10.6 cell line (obtained from NIH AIDS Reagent Program) (52) contain a single integrated latent HIV-GFP reporter genome. ACH2 is a clone of HIV-1 latently infected CD4^+^ CEM cells that contain a single copy of proviral DNA per cell (obtained from NIH AIDS Reagent Program). HeLa-based TZM-bl cells contain an integrated HIV LTR-luciferase construct (obtained from NIH AIDS Reagent Program). Ya is a clone of HIV-1-infected CD4^+^ Jurkat cells that expressed activated HIV-1 (constructed in our lab) (28, 35, 39).

### Cell culture.

C11, J-Lat 10.6, ACH2, Ya, and Jurkat cells were cultured in RPMI 1640 (Gibco; catalog no. C11875500BT) with 10% fetal bovine serum (FBS) (Gibco; catalog no. 10110154) and 1% penicillin/streptomycin (Gibco; catalog no. 15140-122) in a 37°C incubator containing 5% CO_2_. TZM-bl and 293T cells were cultured in Dulbecco’s modified Eagle medium (DMEM) (Gibco; catalog no. C11995500BT) and supplemented with 10% fetal calf serum (Lonsera; catalog no. S711-001S) and 1% penicillin/streptomycin (Gibco) in a 37°C incubator containing 5% CO_2_.

### Antibodies and reagents.

The following antibodies were used throughout this study: anti-FKBP3 from Thermo Fisher (catalog no. PA5-19483); anti-HDAC1 (catalog no. ab280198), anti-HDAC2 (catalog no. ab219053), anti-H3K4ac (catalog no. ab176799), and anti-H3K18ac (catalog no. ab40888) from Abcam (Cambridge, UK); and anti-YY1 (catalog no. 46395) and anti-β-actin (catalog no. 4970) from Cell Signaling Technology (MA, USA). We purchased 2× *Taq* master mix (catalog no. P112) and high-fidelity PCR enzyme 2× Phanta Max master mix (catalog no. P515) from Vazyme (Nanjing, China). PMD18-T (catalog no. 6011) was purchased from TaKaRa (Beijing, China). Cell genome extraction kit (catalog no. DP304) and plasmid extraction kit (catalog nos. DP103, DP108, and DP117) were purchased from Tiangen (Beijing, China). Gel extraction kit (catalog no. CW2302) was purchased from CWBio (Nanjing, China). Luciferase and NanoLuc detection kits (catalog nos. E6110 and N1110, respectively) were purchased from Promega (Madison, USA). Recombinant human IFN-α (catalog no. 11200-1), recombinant human IFN-β (catalog no. 8499-IF), and recombinant human IFN-γ (catalog no. 285-IF) were purchased from R&D Systems (USA). Cell counting kit (CCK-8) and TUNEL apoptosis detection kit (fluorescein isothiocyanate [FITC]) were purchased from Yeasen (Shanghai, China).

### Vector construction.

Individual sgRNA constructs targeting FKBP3 were cloned into lentiCRISPR v2.0 (addgene; catalog no. 52961). For all other experiments, FKBP3-sg1 was used. Individual shRNA constructs targeting FKBP3 were cloned into pLKO.1. For cDNA expression vectors, a linearized lentiviral backbone was generated from pCDH (Youbio, Hunan, China). Protein-coding sequences were a gift from Han Jiahuai’s laboratory. All constructed plasmids were confirmed by restriction enzyme digestion and DNA sequencing.

### Cas9-mediated gene knockout and cDNA overexpression.

C11, J-Lat 10.6, ACH2, and TZM-bl cells were infected with lentivirus at a multiplicity of infection (MOI) of 1 and then selected using 2 μg/ml puromycin for 14 days. Knockout efficiency was analyzed using Sanger DNA sequencing. Knockout efficiency was detected by Western blotting (WB) analysis.

### Visualization of GFP and flow cytometry assay.

Cells were collected and washed with phosphate-buffered saline (PBS). They were kept in PBS before analysis on a BD LSR II flow cytometer for enhanced GFP (EGFP) expression. FlowJo software (FlowJo LLC, Ashland, OR) was used to perform the flow cytometry analysis.

### ELISA detection of antigen p24 levels.

A total of 1 × 10^6^ ACH2 or TZM-bl cells were seeded in a 6-well plate. After 48 h of culture, HIV-1 production was monitored via quantifying the amounts of p24 produced in culture supernatant by using an HIV-1 p24 antigen enzyme-linked immunosorbent assay (ELISA) kit (R&D Systems, MN, USA) according to the manufacturer’s instructions.

### ChIP experiments.

ChIP experiments were performed according to the protocol provided by EZ-ChIP chromatin immunoprecipitation kit (Millipore). Briefly, cells were cross-linked with 1% formaldehyde for 10 min at room temperature and quenched with 0.125 M glycine for 5 min. After lysis, nuclear extracts were separated, and chromatin was sheared by sonicator (Bioruptor UCD-200; Diagenode) for 10 min (10 s on and 10 s off) on ice to obtain DNA fragments of 200 to 1,000 bp in length. One percent of total sheared chromatin DNA was used as the input. Nuclear extracts were incubated with the indicated antibodies at 4°C overnight. Protein G/A-labeled Dynabeads were added to each sample at 4°C for 2 h for immunoprecipitation. The immunoprecipitated DNA was analyzed by a real-time PCR at 40 cycles with Thunderbird SYBR qPCR mix (Toyobo).

### Luciferase reporter assay.

Since the genome of TZM-bl cells was integrated with a luciferase reporter driven by the HIV-1 5′ LTR promoter, TZM-bl cells were used for the luciferase reporter assay. Briefly, cells were harvested at 72 h postinfection, and the lysate was assayed for luciferase activity. Triplicate cultures were measured for each experiment.

### Cell proliferation by CCK-8 assay.

Five thousand cells were added to each well of a 96-well plate. To measure the optical density (OD) value, 10% CCK-8 solution was added to fresh culture medium and incubated at 37°C for 1 h. The OD at 450 nm (OD_450_) value was measured.

### Apoptosis detected by TUNEL staining.

A total of 1 × 10^6^ cells were collected in a 1.5-ml tube and centrifuged at 300 × *g* for 5 min. The cells were washed twice with 500 μl PBS. Cells were treated with TUNEL-FITC apoptosis detection kit according to the manufacturer's instructions. The proportion of FITC-positive cells in the total cells was analyzed by flow cytometry. FlowJo software (FlowJo LLC, Ashland, OR) was used to perform the flow cytometry analysis.

### Western blotting.

A total of 1 × 10^6^ cells were preseeded in a 10-cm dish and cultured 24 h. Then, cells were harvested, lysed, subjected to SDS-PAGE, and then transferred onto nitrocellulose membrane, followed by incubation with indicated primary antibody. Membranes were visualized using the Immun-Star WesternC chemiluminescence kit (Bio-Rad), and images were captured using a ChemiDoc XRS+ system and processed using ImageLab software (Bio-Rad).

### Isolation of primary CD4^+^ T cells.

Peripheral blood mononuclear cells isolated from healthy donors were purchased from the Shanghai Blood Center (Shanghai, China). Naive CD4^+^ T cells were further purified from peripheral blood mononuclear cells by negative selection according to the manufacturer's instructions (Thermo Fisher). The naive CD4^+^ T cells were maintained in serum-free medium supplemented with 1% penicillin-streptomycin, 5 ng/ml recombinant human interleukin-2 (R&D Systems), and 10 ng/ml recombinant human interleukin-7 (R&D Systems) at 37°C under 5% CO_2_.

### Statistical analysis.

Data are representative of three independent experiments, and error bars represent standard errors (SD). Paired-sample *t* tests were performed with use of SPSS, version 13.0 (SPSS Inc., Chicago), and statistical significance was indicated at *, *P < *0.05; **, *P < *0.01; and ***, *P < *0.001.

## References

[1] Ruelas DS, Greene WC. 2013. An integrated overview of HIV-1 latency. Cell 155:519–529. doi:10.1016/j.cell.2013.09.044. 24243012 PMC4361081

[2] Margolis DM, Garcia JV, Hazuda DJ, Haynes BF. 2016. Latency reversal and viral clearance to cure HIV-1. Science 353:aaf6517. doi:10.1126/science.aaf6517. 27463679 PMC5021637

[3] Davey RT, Bhat N, Yoder C, Chun TW, Metcalf JA, Dewar R, Natarajan V, Lempicki RA, Adelsberger JW, Miller KD, Kovacs JA, Polis MA, Walker RE, Falloon J, Masur H, Gee D, Baseler M, Dimitrov DS, Fauci AS, Lane HC. 1999. HIV-1 and T cell dynamics after interruption of highly active antiretroviral therapy (HAART) in patients with a history of sustained viral suppression. Proc Natl Acad Sci U S A 96:15109–15114. doi:10.1073/pnas.96.26.15109. 10611346 PMC24781

[4] Dahabieh MS, Battivelli E, Verdin E. 2015. Understanding HIV latency: the road to an HIV cure. Annu Rev Med 66:407–421. doi:10.1146/annurev-med-092112-152941. 25587657 PMC4381961

[5] Finzi D, Hermankova M, Pierson T, Carruth LM, Buck C, Chaisson RE, Quinn TC, Chadwick K, Margolick J, Brookmeyer R, Gallant J, Markowitz M, Ho DD, Richman DD, Siliciano RF. 1997. Identification of a reservoir for HIV-1 in patients on highly active antiretroviral therapy. Science 278:1295–1300. doi:10.1126/science.278.5341.1295. 9360927

[6] Chun TW, Engel D, Berrey MM, Shea T, Corey L, Fauci AS. 1998. Early establishment of a pool of latently infected, resting CD4(+) T cells during primary HIV-1 infection. Proc Natl Acad Sci U S A 95:8869–8873. doi:10.1073/pnas.95.15.8869. 9671771 PMC21169

[7] Barouch DH, Deeks SG. 2014. Immunologic strategies for HIV-1 remission and eradication. Science 345:169–174. doi:10.1126/science.1255512. 25013067 PMC4096716

[8] Verdin E, Paras P, Van LC. 1993. Chromatin disruption in the promoter of human immunodeficiency virus type 1 during transcriptional activation. EMBO J 12:3249–3259. doi:10.1002/j.1460-2075.1993.tb05994.x. 8344262 PMC413592

[9] Bieniasz PD, Grdina TA, Bogerd HP, Cullen BR. 1999. Recruitment of cyclin T1/P-TEFb to an HIV type 1 long terminal repeat promoter proximal RNA target is both necessary and sufficient for full activation of transcription. Proc Natl Acad Sci U S A 96:7791–7796. doi:10.1073/pnas.96.14.7791. 10393900 PMC22140

[10] Blazkova J, Trejbalova K, Gondois-Rey F, Halfon P, Philibert P, Guiguen A, Verdin E, Olive D, Van Lint C, Hejnar J, Hirsch I. 2009. CpG methylation controls reactivation of HIV from latency. PLoS Pathog 5:e1000554. doi:10.1371/journal.ppat.1000554. 19696893 PMC2722084

[11] Archin NM, Sung JM, Garrido C, Soriano-Sarabia N, Margolis DM. 2014. Eradicating HIV-1 infection: seeking to clear a persistent pathogen. Nat Rev Microbiol 12:750–764. doi:10.1038/nrmicro3352. 25402363 PMC4383747

[12] Kumar A, Darcis G, Van Lint C, Herbein G. 2015. Epigenetic control of HIV-1 post integration latency: implications for therapy. Clin Epigenetics 7:103. doi:10.1186/s13148-015-0137-6. 26405463 PMC4581042

[13] Elsheikh MM, Tang Y, Li D, Jiang G. 2019. Deep latency: a new insight into a functional HIV cure. EBioMedicine 45:624–629. doi:10.1016/j.ebiom.2019.06.020. 31227439 PMC6642357

[14] Friedman J, Cho W-K, Chu CK, Keedy KS, Archin NM, Margolis DM, Karn J. 2011. Epigenetic silencing of HIV-1 by the histone H3 lysine 27 methyltransferase enhancer of zeste 2. J Virol 85:9078–9089. doi:10.1128/JVI.00836-11. 21715480 PMC3165831

[15] Nguyen K, Das B, Dobrowolski C, Karn J. 2017. Multiple histone lysine methyltransferases are required for the establishment and maintenance of HIV-1 latency. mBio 8:e00133-17. doi:10.1128/mBio.00133-17. 28246360 PMC5347344

[16] Lusic M, Marcello A, Cereseto A, Giacca M. 2003. Regulation of HIV-1 gene expression by histone acetylation and factor recruitment at the LTR promoter. EMBO J 22:6550–6561. doi:10.1093/emboj/cdg631. 14657027 PMC291826

[17] Zhang H-S, Ruan Z, Sang W-W. 2011. HDAC1/NFκB pathway is involved in curcumin inhibiting of Tat‐mediated long terminal repeat transactivation. J Cell Physiol 226:3385–3391. doi:10.1002/jcp.22691. 21344388

[18] Coull JJ, Romerio F, Sun JM, Volker JL, Galvin KM, Davie JR, Shi Y, Hansen U, Margolis DM. 2000. The human factors YY1 and LSF repress the human immunodeficiency virus type 1 long terminal repeat via recruitment of histone deacetylase 1. J Virol 74:6790–6799. doi:10.1128/jvi.74.15.6790-6799.2000. 10888618 PMC112196

[19] du Chéné I, Basyuk E, Lin Y-L, Triboulet R, Knezevich A, Chable-Bessia C, Mettling C, Baillat V, Reynes J, Corbeau P, Bertrand E, Marcello A, Emiliani S, Kiernan R, Benkirane M. 2007. Suv39H1 and HP1γ are responsible for chromatin-mediated HIV-1 transcriptional silencing and post-integration latency. EMBO J 26:424–435. doi:10.1038/sj.emboj.7601517. 17245432 PMC1783455

[20] Fiume G, Vecchio E, De Laurentiis A, Trimboli F, Palmieri C, Pisano A, Falcone C, Pontoriero M, Rossi A, Scialdone A, Fasanella Masci F, Scala G, Quinto I. 2012. Human immunodeficiency virus-1 Tat activates NF-kB via physical interaction with IkBα and p65. Nucleic Acids Res 40:3548–3562. doi:10.1093/nar/gkr1224. 22187158 PMC3333881

[21] Williams SA, Chen L-F, Kwon H, Ruiz-Jarabo CM, Verdin E, Greene WC. 2006. NF-kB p50 promotes HIV latency through HDAC recruitment and repression of transcriptional initiation. EMBO J 25:139–149. doi:10.1038/sj.emboj.7600900. 16319923 PMC1356344

[22] Sgarbanti M, Remoli AL, Marsili G, Ridolfi B, Borsetti A, Perrotti E, Orsatti R, Ilari R, Sernicola L, Stellacci E, Ensoli B, Battistini A. 2008. IRF-1 is required for full NF-kB transcriptional activity at the human immunodeficiency virus type 1 long terminal repeat enhancer. J Virol 82:3632–3641. doi:10.1128/JVI.00599-07. 18216101 PMC2268499

[23] Zhu Y, Pe'ery T, Peng J, Ramanathan Y, Marshall N, Marshall T, Amendt B, Mathews MB, Price DH. 1997. Transcription elongation factor P-TEFb is required for HIV-1 Tat transactivation in vitro. Genes Dev 11:2622–2632. doi:10.1101/gad.11.20.2622. 9334325 PMC316609

[24] Bieniasz PD, Grdina TA, Bogerd HP, Cullen BR. 1998. Recruitment of a protein complex containing Tat and cyclin T1 to TAR governs the species specificity of HIV-1 Tat. EMBO J 17:7056–7065. doi:10.1093/emboj/17.23.7056. 9843510 PMC1171053

[25] Lu H, Li Z, Zhang W, Schulze-Gahmen U, Xue Y, Zhou Q. 2015. Gene target specificity of the super elongation complex (SEC) family: how HIV-1 Tat employs selected SEC members to activate viral transcription. Nucleic Acids Res 43:5868–5879. doi:10.1093/nar/gkv541. 26007649 PMC4499153

[26] Ruelas DS, Chan JK, Oh E, Heidersbach AJ, Hebbeler AM, Chavez L, Verdin E, Rape M, Greene WC. 2015. MicroRNA-155 reinforces HIV latency. J Biol Chem 290:13736–13748. doi:10.1074/jbc.M115.641837. 25873391 PMC4447952

[27] Kula A, Marcello A. 2012. Dynamic post-transcriptional regulation of HIV-1 gene expression. Biology (Basel) 1:116–133. doi:10.3390/biology1020116. 24832221 PMC4009772

[28] Wang P, Qu X, Zhou X, Shen Y, Ji H, Fu Z, Deng J, Lu P, Yu W, Lu H, Zhu H. 2015. Two cellular microRNAs, miR-196b and miR-1290, contribute to HIV-1 latency. Virology 486:228–238. doi:10.1016/j.virol.2015.09.016. 26469550

[29] Archin NM, Liberty AL, Kashuba AD, Choudhary SK, Kuruc JD, Crooks AM, Parker DC, Anderson EM, Kearney MF, Strain MC, Richman DD, Hudgens MG, Bosch RJ, Coffin JM, Eron JJ, Hazuda DJ, Margolis DM. 2012. Administration of vorinostat disrupts HIV-1 latency in patients on antiretroviral therapy. Nature 487:482–485. doi:10.1038/nature11286. 22837004 PMC3704185

[30] Søgaard OS, Graversen ME, Leth S, Olesen R, Brinkmann CR, Nissen SK, Kjaer AS, Schleimann MH, Denton PW, Hey-Cunningham WJ, Koelsch KK, Pantaleo G, Krogsgaard K, Sommerfelt M, Fromentin R, Chomont N, Rasmussen TA, Østergaard L, Tolstrup M. 2015. The depsipeptide romidepsin reverses HIV-1 latency in vivo. PLoS Pathog 11:e1005142. doi:10.1371/journal.ppat.1005142. 26379282 PMC4575032

[31] Cong L, Ran FA, Cox D, Lin S, Barretto R, Habib N, Hsu PD, Wu X, Jiang W, Marraffini LA, Zhang F. 2013. Multiplex genome engineering using CRISPR/Cas systems. Science 339:819–823. doi:10.1126/science.1231143. 23287718 PMC3795411

[32] Hsu PD, Lander ES, Zhang F. 2014. Development and applications of CRISPR-Cas9 for genome engineering. Cell 157:1262–1278. doi:10.1016/j.cell.2014.05.010. 24906146 PMC4343198

[33] Wang T, Wei JJ, Sabatini DM, Lander ES. 2014. Genetic screens in human cells using the CRISPR-Cas9 system. Science 343:80–84. doi:10.1126/science.1246981. 24336569 PMC3972032

[34] Shalem O, Sanjana NE, Hartenian E, Shi X, Scott DA, Mikkelson T, Heckl D, Ebert BL, Root DE, Doench JG, Zhang F. 2014. Genome-scale CRISPR-Cas9 knockout screening in human cells. Science 343:84–87. doi:10.1126/science.1247005. 24336571 PMC4089965

[35] Xinyi Y, Yanan W, Panpan L, Yinzhong S, Xiaying Z, Yuqi Z, Zhengtao J, He Y, Hanyu P, Lin Z, Yangcheng Z, Jing W, Zhiming L, Xiaoting S, Daru L, Shibo J, Jianqing X, Hao W, Hongzhou L, Guochun J, Huanzhang Z. 2020. PEBP1 suppresses HIV transcription and induces latency by inactivating MAPK/NF-κB signaling. EMBO Rep 21:e49305. doi:10.15252/embr.201949305. 32924251 PMC7645261

[36] Breiman A, Camus I. 2002. The involvement of mammalian and plant FK506-binding proteins (FKBPs) in development. Transgenic Res 11:321–335. doi:10.1023/a:1016331814412. 12212836

[37] Jin YJ, Burakoff SJ, Bierer BE. 1992. Molecular cloning of a 25-kDa high affinity rapamycin binding protein, FKBP25. J Biol Chem 267:10942–10945. doi:10.1016/S0021-9258(19)49856-6. 1375932

[38] Ochocka AM, Kampanis P, Nicol S, Allende-Vega N, Cox M, Marcar L, Milne D, Fuller-Pace F, Meek D. 2009. FKBP25, a novel regulator of the p53 pathway, induces the degradation of MDM2 and activation of p53. FEBS Lett 583:621–626. doi:10.1016/j.febslet.2009.01.009. 19166840

[39] Qu X, Wang P, Ding D, Li L, Wang H, Ma L, Zhou X, Liu S, Lin SG, Wang X, Zhang G, Liu SJ, Liu L, Wang J, Zhang F, Lu D, Zhu H. 2013. Zinc-finger-nucleases mediate specific and efficient excision of HIV-1 proviral DNA from infected and latently infected human T cells. Nucleic Acids Res 41:7771–7782. doi:10.1093/nar/gkt571. 23804764 PMC3763554

[40] Yang X, Zhu X, Ji H, Deng J, Lu P, Jiang Z, Li X, Wang Y, Wang C, Zhao J, Wang Y, Zhong Y, Yang H, Zhu H. 2018. Quercetin synergistically reactivates human immunodeficiency virus type 1 latency by activating nuclear factor-kB. Mol Med Rep 17:2501–2508. doi:10.3892/mmr.2017.8188. 29207194

[41] Yang WM, Yao YL, Seto E. 2001. The FK506-binding protein 25 functionally associates with histone deacetylases and with transcription factor YY1. EMBO J 20:4814–4825. doi:10.1093/emboj/20.17.4814. 11532945 PMC125595

[42] Wightman F, Ellenberg P, Churchill M, Lewin SR. 2012. HDAC inhibitors in HIV. Immunol Cell Biol 90:47–54. doi:10.1038/icb.2011.95. 22083528

[43] Hao Y, Zhang Y, Zhou X, Qu X, Wang P, Liu S, Lu D, Lu H, Zhu H. 2012. Selective histonedeacetylase inhibitor M344 intervenes in HIV-1 latency through increasing histone acetylation and activation of NF-kappaB. PLoS One 7:e48832. doi:10.1371/journal.pone.0048832. 23166597 PMC3499534

[44] Yang W, Sun Z, Hua C, Wang Q, Xu W, Deng Q, Pan Y, Lu L, Jiang S. 2018. Chidamide, a histone deacetylase inhibitor-based anticancer drug, effectively reactivates latent HIV-1 provirus. Microbes Infect 20:626–634. doi:10.1016/j.micinf.2017.10.003. 29126877

[45] Bosque A, Planelles V. 2011. Studies of HIV-1 latency in an ex vivo model that uses primary central memory T cells. Methods 53:54–61. doi:10.1016/j.ymeth.2010.10.002. 20970502 PMC3031099

[46] Kim M, Hosmane NN, Bullen CK, Capoferri A, Yang H-C, Siliciano JD, Siliciano RF. 2014. A primary CD4(+) T cell model of HIV-1 latency established after activation through the T cell receptor and subsequent return to quiescence. Nat Protoc 9:2755–2770. doi:10.1038/nprot.2014.188. 25375990 PMC4378543

[47] Hotter D, Bosso M, Jønsson KL, Krapp C, Stürzel CM, Das A, Littwitz-Salomon E, Berkhout B, Russ A, Wittmann S, Gramberg T, Zheng Y, Martins LJ, Planelles V, Jakobsen MR, Hahn BH, Dittmer U, Sauter D, Kirchhoff F. 2019. IFI16 targets the transcription factor Sp1 to suppress HIV-1 transcription and latency reactivation. Cell Host Microbe 25:858–872. doi:10.1016/j.chom.2019.05.002. 31175045 PMC6681451

[48] Liu Y, Fu Y, Wang Q, Li M, Zhou Z, Dabbagh D, Fu C, Zhang H, Li S, Zhang T, Gong J, Kong X, Zhai W, Su J, Sun J, Zhang Y, Yu X-F, Shao Z, Zhou F, Wu Y, Tan X. 2019. Proteomic profiling of HIV-1 infection of human CD4+ T cells identifies PSGL-1 as an HIV restriction factor. Nat Microbiol 4:813–825. doi:10.1038/s41564-019-0372-2. 30833724

[49] Nasr AB, Ponnala D, Sagurthi SR, Kattamuri RK, Marri VK, Gudala S, Lakkaraju C, Bandaru S, Nayarisseri A. 2015. Molecular docking studies of FKBP12-mTOR inhibitors using binding predictions. Bioinformation 11:307–315. doi:10.6026/97320630011307. 26229292 PMC4512006

[50] Wang X, Fonseca BD, Tang H, Liu R, Elia A, Clemens MJ, Bommer U-A, Proud CG. 2008. Re-evaluating the roles of proposed modulators of mammalian target of rapamycin complex 1 (mTORC1) signaling. J Biol Chem 283:30482–30492. doi:10.1074/jbc.M803348200. 18676370 PMC2662142

[51] Hausch F, Kozany C, Theodoropoulou M, Fabian A-K. 2013. FKBPs and the Akt/mTOR pathway. Cell Cycle 12:2366–2370. doi:10.4161/cc.25508. 23839048 PMC3841315

[52] Jordan A, Defechereux P, Verdin E. 2001. The site of HIV-1 integration in the human genome determines basal transcriptional activity and response to Tat transactivation. EMBO J 20:1726–1738. doi:10.1093/emboj/20.7.1726. 11285236 PMC145503

