# Supplementary figures and images for: FKBP3 Induces Human Immunodeficiency Virus Type 1 Latency by Recruiting Histone Deacetylase 1/2 to the Viral Long Terminal Repeat
Source: mBio. 2021 Jul 20;12(4):e00795-21. doi: 10.1128/mBio.00795-21 (PMC8406261; doi:10.1128/mBio.00795-21)

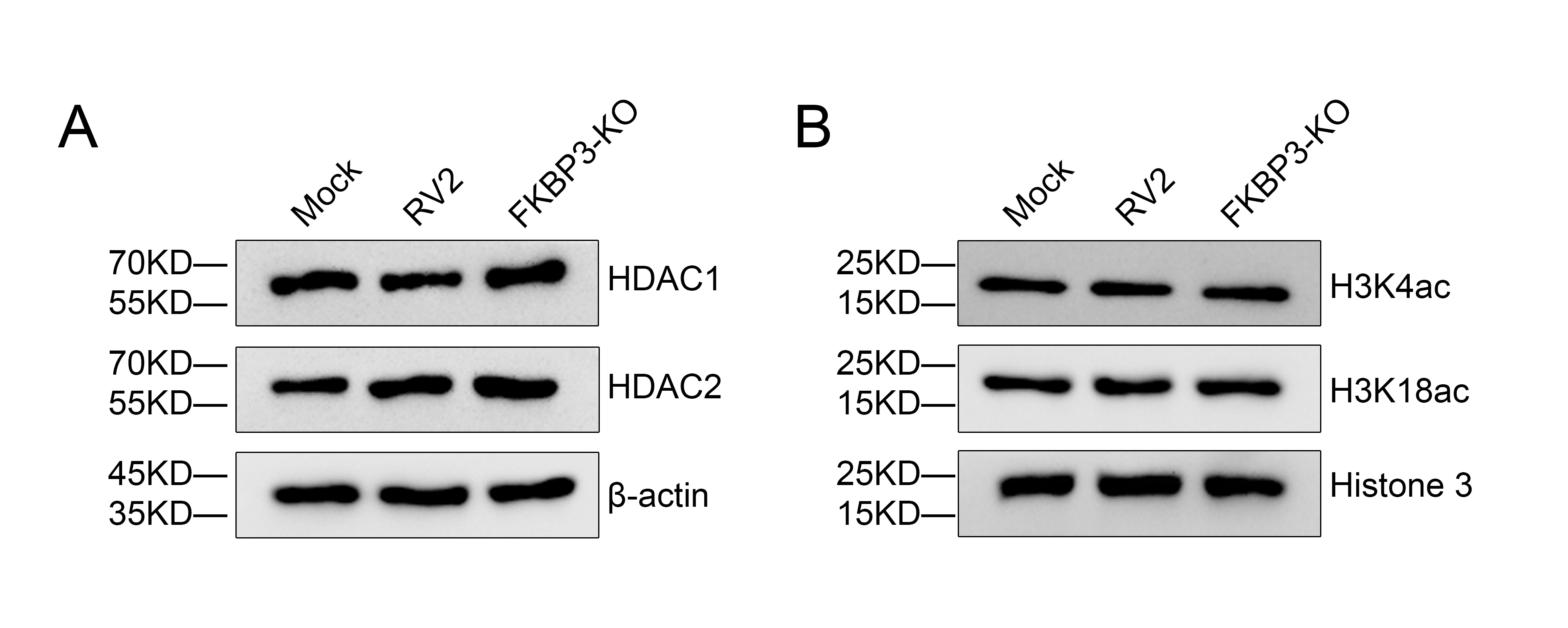

Supplement: FIG S1 [file mbio.00795-21-sf001.tif]

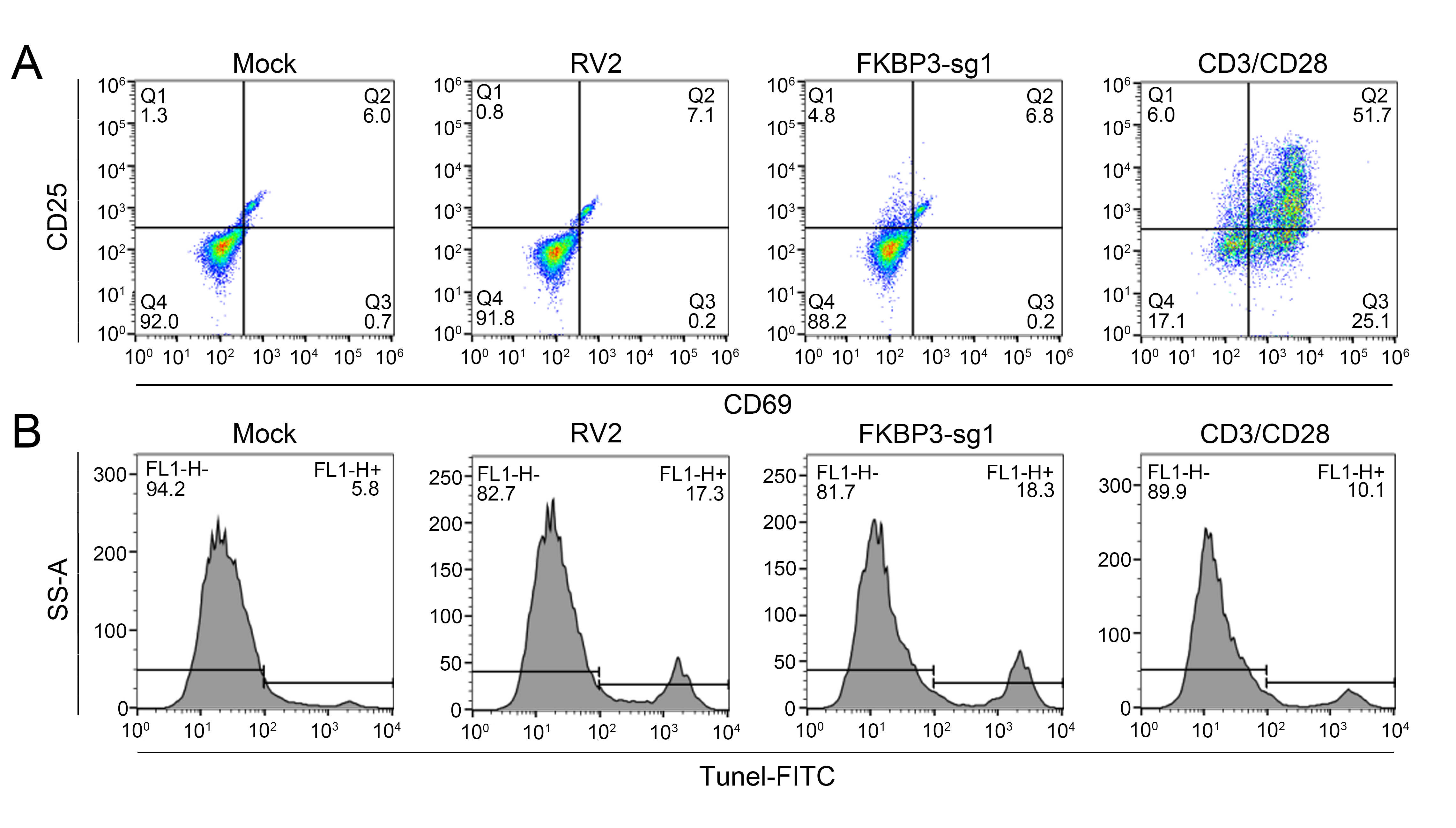

Supplement: FIG S2 [file mbio.00795-21-sf002.tif]

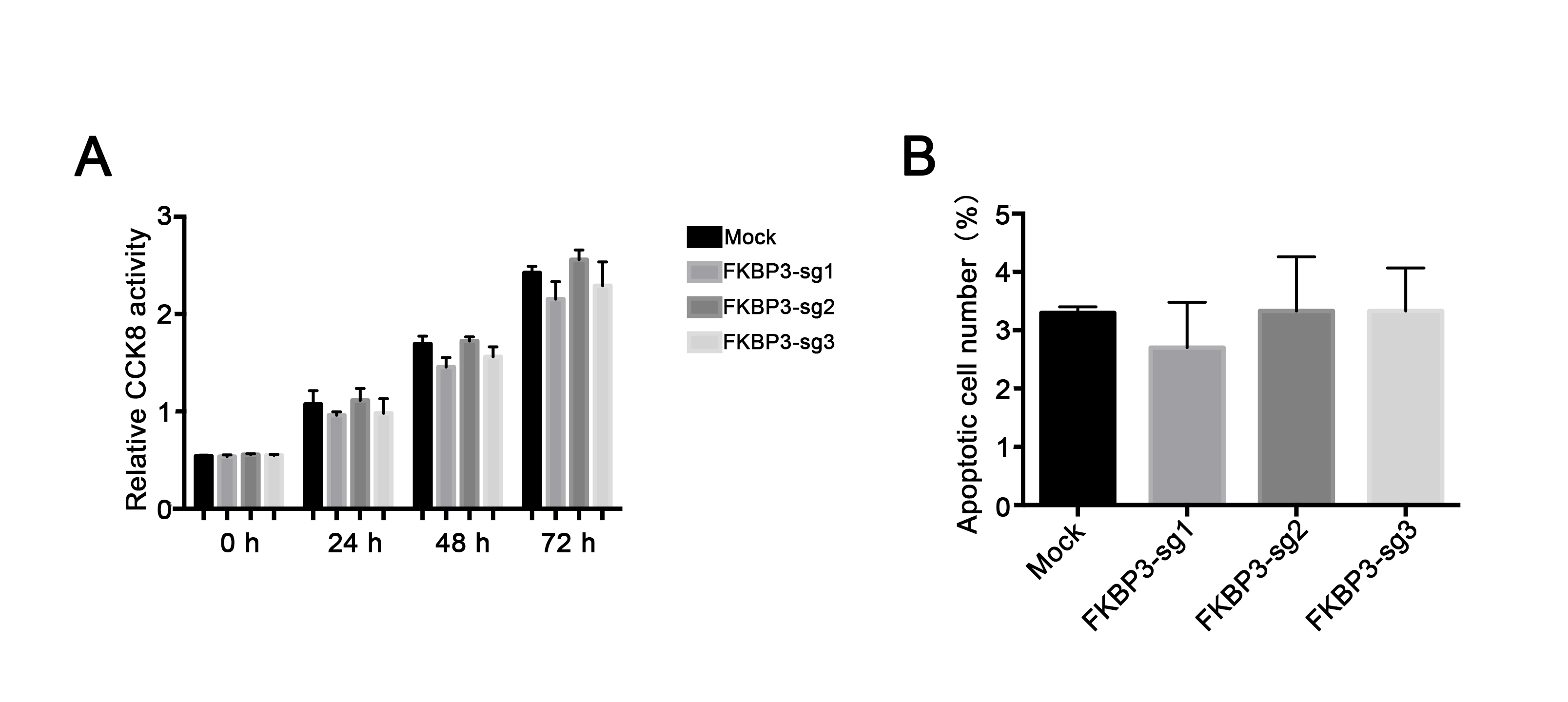

Supplement: FIG S3 [file mbio.00795-21-sf003.tif]
